# Association of serum albumin levels with multidomain functional impairment (motor, balance, ADL, and cognitive impairment) in Chinese post-stroke patients: a multicenter cross-sectional study

**DOI:** 10.3389/fneur.2026.1763978

**Published:** 2026-02-19

**Authors:** Jie Zhu, Ranran Bi, Yansheng Lin, Shuyang Zhang, Yifang Lin, Xuezhen Zhao, Jiali Lin, Jie Jia

**Affiliations:** 1 Department of Rehabilitation Medicine, Huashan Hospital, Fudan University, Shanghai, China; 2 Department of Rehabilitation Medicine, Shanghai East Hospital, School of Medicine, Tongji University, Shanghai, China; 3 Department of Rehabilitation Medicine, Fujian University of Traditional Chinese Medicine, Fuzhou, China; 4 Department of Rehabilitation Medicine, The First Affiliated Hospital of Fujian Medical University, Fuzhou, China; 5 National Clinical Research Center for Aging and Medicine, Huashan Hospital, Fudan University, Shanghai, China

**Keywords:** activities of daily living (ADL), Fugl-Meyer assessment (FMA), functional recovery, serum albumin, stroke rehabilitation

## Abstract

**Background:**

Malnutrition, frequently indicated by hypoalbuminemia, is prevalent post-stroke and associated with adverse functional outcomes. However, the independent role of serum albumin (ALB) in multidomain functional recovery—encompassing motor, balance, cognitive, and daily living domains—remains underexplored in Chinese populations. This multicenter study aimed to quantify the independent association between serum ALB levels and functional impairment in Chinese post-stroke patients.

**Methods:**

In this cross-sectional study, 1,741 patients from rehabilitation centers across China were enrolled. ALB levels were categorized into quartiles (Q1: <37.7 g/L; Q2: 37.7–40.0 g/L; Q3: 40.0–42.8 g/L; Q4: ≥42.8 g/L). Outcomes included motor function (Fugl-Meyer Assessment), activities of daily living (Modified Barthel Index), balance (Berg Balance Scale), and cognition (Montreal Cognitive Assessment). Multivariable linear regression models adjusted for demographics, comorbidities, lesion characteristics, and illness duration. Subgroup analyses tested interactions by age, sex, BMI, and lesion topography.

**Results:**

Each 1-g/L ALB increase independently predicted functional gains: FMA (*β* = 1.35, 95% CI: 0.99–1.72), ADL (β = 1.77, 1.44–2.10), BBS (β = 1.02, 0.78–1.26), MoCA (β = 0.30, 0.21–0.40) (all *p* < 0.001). Dose-dependent improvements were observed across quartiles (Q4 vs. Q1: FMA Δβ = 15.11 [11.09–19.12]; ADL Δβ = 19.35[15.76–22.93]; P trend < 0.001). Sex significantly modified ALB-FMA associations (P interaction = 0.017), with females showing stronger effects (*β* = 1.81 [1.12–2.51]) than males (β = 1.15 [0.72–1.58]). Cerebellar lesions demonstrated non-significant trend toward amplified associations (FMA: β = 2.16 [0.72–3.59]).

**Conclusion:**

ALB levels are independently and dose-dependently associated with motor, ADL, balance, and cognitive function in post-stroke patients. Compared to lower quartiles, patients with ALB ≥42.8 g/L (highest quartile) exhibit superior functional outcomes. A sex-specific pattern is observed solely in motor function, where the correlation is more pronounced in females. ALB may serve as a biological indicator for risk stratification during stroke rehabilitation.

## Introduction

Stroke remains a leading cause of long-term disability worldwide, imposing a substantial socioeconomic burden, particularly in China, which harbors the highest number of stroke survivors globally (1–3). Post-stroke functional impairment is multifaceted, spanning deficits in motor control, balance, activities of daily living, and cognition (4–6). Malnutrition, prevalent in stroke patients, exacerbates poor outcomes, with serum albumin (ALB) serving as a widely used, cost-effective biomarker of nutritional status and systemic inflammation (7–9).

Current literature consistently links hypoalbuminemia to unfavorable prognoses, such as increased mortality and complications (10, 11). However, existing research is often fragmented, primarily focusing on the association between low ALB and single-domain outcomes like mortality or gross physical disability (11, 12). Critically, a significant gap remains regarding the comprehensive impact of ALB status on multidomain functional impairment—specifically, its simultaneous and independent association with objective, integrated measures of motor, balance, ADL, and detailed cognitive function. Addressing this deficiency, the purpose of this large-scale, multicenter cross-sectional study was to systematically investigate the independent and dose-dependent association of serum ALB levels with comprehensive functional impairment (motor, balance, ADL, and cognition) in Chinese post-stroke patients.

## Methods

### Study design and participants

**Figure 1:**
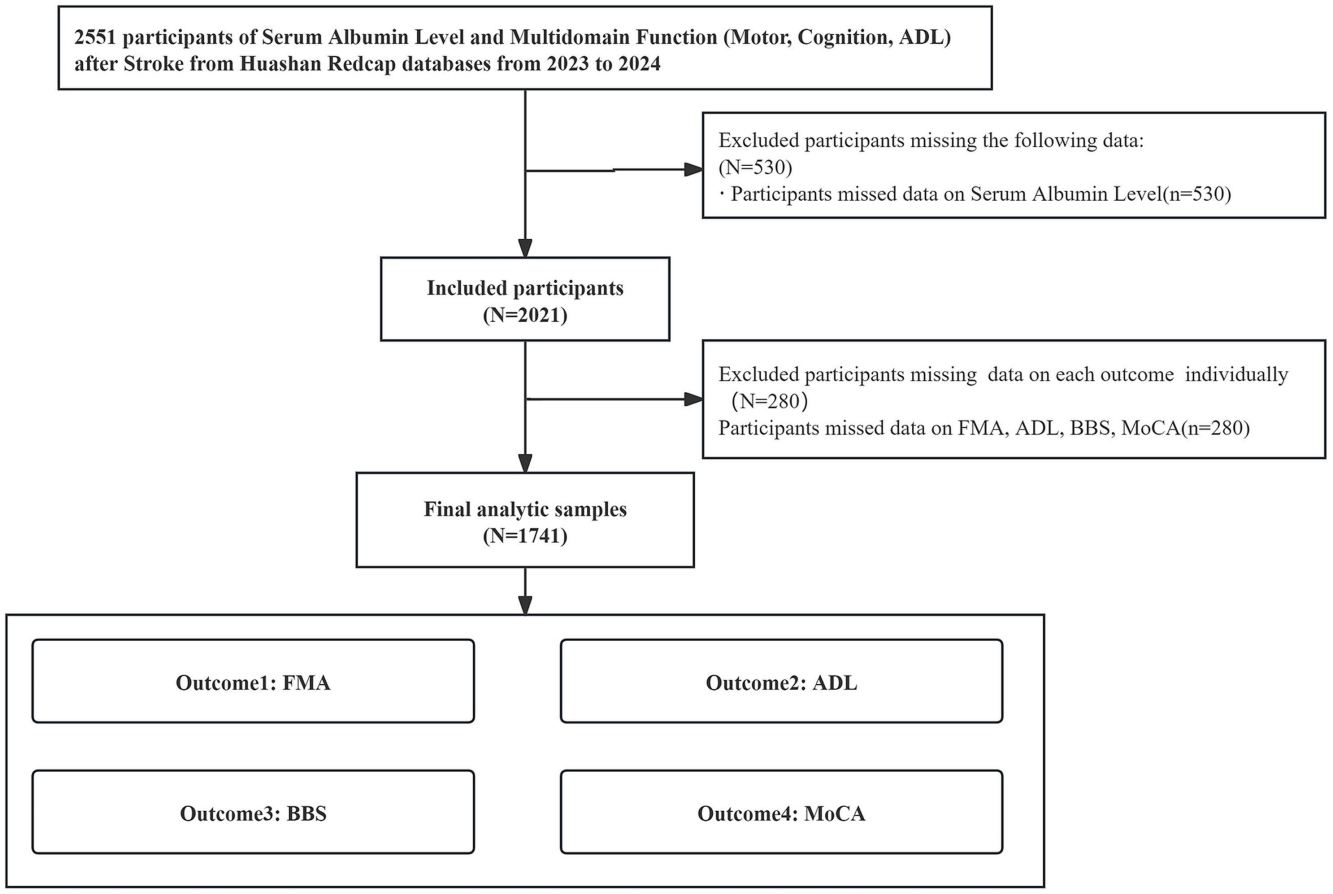
Flowchart of participant inclusion.

This multicenter cross-sectional study was conducted across 26 tertiary rehabilitation centers in China between January 2023 and December 2024 (Figure 1). The study protocol was approved by the Ethics Review Committee of Huashan Hospital, Fudan University (approval number: HIRB2022-510). The study was registered in the Chinese Clinical Trial Registry (registration number: ChiCTR2200063611). Written informed consent was obtained from all participants or their legal representatives. The study adhered to the Declaration of Helsinki and STROBE guidelines for observational studies (13).

### Participant recruitment and data management

A total of 1,741 adults (≥18 years) with clinically confirmed ischemic or hemorrhagic stroke were enrolled. Patients were excluded based on clinical conditions that could substantially confound the assessment of functional recovery or precipitate clinical deterioration during evaluation, including: (1) Acute Uncontrolled Medical Conditions: Severe or poorly controlled hypertension, advanced heart failure, severe active infection (e.g., pneumonia), diabetic ketoacidosis, uncontrolled or frequent seizures, or other acute medical problems judged by the treating clinician as likely to worsen during assessment. (2) Uncontrolled Systemic Disorders: Overt thyroid dysfunction (hyper- or hypothyroidism), severe hepatic or renal impairment (including dialysis dependence), active acute rheumatologic disease, or clinically significant electrolyte imbalances. (3) Data Quality: Records with missing key covariates or outcomes, implausible values, or duplicate entries were excluded during the final data cleaning process, yielding the final analytic cohort.

All clinical and demographic data were collected and managed using a secure, electronic data capture platform (REDCap; https://himedc.huashan.org.cn:5288/) hosted at the Huashan Hospital, Fudan University coordinating center, ensuring data privacy and quality control across all participating sites.

### Assessment of exposure and outcome variables

#### Exposure variable

The primary exposure variable was ALB level (g/L), measured from venous blood samples collected upon admission or within 48 h of functional assessment, using standard automated laboratory methods. For analysis, ALB was treated as a continuous variable (per 1 g/L increment) and a categorical variable (stratified into quartiles Q1:<37.7; Q2:37.7–40.0; Q3:40.0–42.8; Q4:≥42.8) (14).

#### Outcome variables

The four primary functional outcome variables were assessed by trained therapists or neuropsychologists using standardized protocols: (1) Motor Function: Assessed using the FMA of the paretic upper and lower extremities (total score, 0–100), where higher scores indicate better motor recovery (15). (2) Activities of Daily Living (ADL): Assessed using the Modified Barthel Index (MBI) (total score, 0–100), where higher scores indicate greater independence. (3) Balance function: assessed using the Berg Balance Scale (BBS) (total score, 0–56), where higher scores indicate better balance performance (16). (4) Cognitive function: assessed using the Montreal Cognitive Assessment (MoCA) (total score, 0–30), where higher scores indicate better global cognitive status. Based on established guidelines, MoCA scores were adjusted for education level (17–19).

### Covariates

Potential confounding variables were collected to ensure comprehensive adjustment in the multiple linear regression models. These covariates included Demographic and Lifestyle Factors (Sex, Age, Education level, Body Mass Index [BMI], Smoking status, and Alcohol status), Comorbidities (History of Hypertension, Coronary Artery Disease [CAD], Diabetes Mellitus [DM], and Previous stroke history), and Stroke Characteristics. The detailed stroke characteristics encompassed Day_of_illness (time span in days from stroke onset to functional assessment), Stroke diagnosis (Ischemic vs. Hemorrhagic), and Lesion location (lesions involving the Basal Ganglia[BG], Brainstem[BS], and Cerebellum[CB]) as confirmed by neuroimaging reports.

### Statistical analyses

Participants were stratified into quartiles based on ALB levels: Q1 (<37.7 g/L), Q2 (37.7–40.0 g/L), Q3 (40.0–42.8 g/L), and Q4 (≥42.8 g/L). Descriptive statistics are presented as mean ± standard deviation (SD) for normally distributed continuous variables, median (interquartile range) for skewed data, and frequency (%) for categorical variables. Group comparisons were performed using ANOVA or Kruskal-Wallis tests for continuous variables and χ^2^ tests for categorical variables, as appropriate. Statistical significance was set at *p* < 0.05 (two-tailed).

Multivariable linear regression models assessed associations between ALB levels and functional outcomes (FMA, ADL, BBS, MoCA). Three models were constructed: Model 1 Unadjusted; Model 2: Adjusted for sex, age, education, smoking, alcohol status, BMI, hypertension diabetes mellitus (DM), coronary artery disease (CAD), and previous stroke; Model 3: Further adjusted for Model 2 covariates plus illness duration (days) and lesion locations (BG, BS, CB). ALB was analyzed continuously (per 1-g/L increase) and categorically (quartiles: <37.7 g/L [reference], 37.7–40.0 g/L, 40.0–42.8 g/L, ≥42.8 g/L). Results are presented as regression coefficients (*β*) with 95% confidence intervals (CI). Linear trend tests across quartiles were performed. Significance was defined as *p* < 0.05.

Subgroup analyses were conducted using stratified multivariable linear regression models to assess the heterogeneity of associations between ALB and functional outcomes (FMA, ADL, BBS, MoCA) across prespecified subgroups. Subgroups included age (<60 vs. ≥60 years), sex (male vs. female), BMI (<24 vs. ≥24 kg/m^2^), and lesion locations (BG, BS, CB). Interaction terms (ALB × subgroup) were tested, with significance defined as *p* < 0.05. Results are presented as *β*-coefficients per 1-g/L ALB increase with 95% confidence intervals (CI).

## Results

### Baseline characteristics of study participants

The baseline characteristics of the 1,741 post-stroke patients, categorized by quartiles of ALB (Q1: < 37.7 g/L to Q4: ≥42.8 g/L), are presented in Table 1. Significant differences across the albumin quartiles were observed for Sex (*p* < 0.001), with males constituting a larger proportion in the highest quartile (77%). Continuous variables, including Age (*p* < 0.001), BMI (*p* < 0.001), and ALB itself (*p* < 0.001), also differed significantly across the groups. Notably, the mean age decreased progressively from Q1 (65.7 ± 11.2 years) to Q4 (57.0 ± 13.2 years). Significant differences were also found in the prevalence of Hypertension (*p* = 0.04) and Diabetes Mellitus (DM) (*p* = 0.012), with the highest prevalence of DM (13.9%) recorded in Q4. Furthermore, Day_of_illness differed significantly (*p* < 0.001), showing the longest median duration in Q1 (36.0 days) and the shortest in Q4 (23.0 days). Education, smoking, alcohol use, and comorbidities (CAD, stroke history, ischemic stroke) did not differ significantly among groups. All four primary functional outcome scores—FMA, ADL, BBS, and MoCA—showed significant differences across the quartiles (*p* < 0.001 for all), with mean or median scores consistently increasing from the lowest albumin quartile (Q1) to the highest (Q4).

**Table 1:** Baseline characteristics of study participants.

Variables	Total (*N* = 1741)	Q1 37.7 < g/L (*N* = 435)	Q2 37.7–40.0 g/L (*N* = 414)	Q3 42–42.8 g/L (*N* = 454)	Q4 ≥ 42.8 g/L (*N* = 439)	*P*
Sex, *n* (%)						< 0.001
Male	1,210 (69.5)	286 (65.7)	271 (65.6)	315 (69.4)	338 (77)	
Female	531 (30.5)	149 (34.3)	142 (34.4)	139 (30.6)	101 (23)	
Age, years	61.5 ± 12.5	65.7 ± 11.2	63.5 ± 11.7	60.0 ± 11.9	57.0 ± 13.2	< 0.001
BMI, kg/m^2^	23.9 ± 3.1	23.4 ± 3.2	23.9 ± 3.0	24.2 ± 3.1	24.3 ± 3.0	< 0.001
Education, *n* (%)						0.302
Below high school	1,147 (65.9)	294 (67.6)	280 (67.8)	302 (66.5)	271 (61.7)	
High school graduate	337 (19.4)	78 (17.9)	83 (20.1)	86 (18.9)	90 (20.5)	
College or above	257 (14.8)	63 (14.5)	50 (12.1)	66 (14.5)	78 (17.8)	
Smoke, *n* (%)						0.229
No	1,216 (69.8)	314 (72.2)	298 (72.2)	309 (68.1)	295 (67.2)	
Yes	525 (30.2)	121 (27.8)	115 (27.8)	145 (31.9)	144 (32.8)	
Drink, *n* (%)						0.171
No	1,451 (83.3)	365 (83.9)	357 (86.4)	373 (82.2)	356 (81.1)	
Yes	290 (16.7)	70 (16.1)	56 (13.6)	81 (17.8)	83 (18.9)	
Hypertension, *n* (%)						0.04
No	642 (36.9)	182 (41.8)	157 (38)	157 (34.6)	146 (33.3)	
Yes	1,099 (63.1)	253 (58.2)	256 (62)	297 (65.4)	293 (66.7)	
CAD, *n* (%)						0.148
No	1,659 (95.3)	406 (93.3)	394 (95.4)	437 (96.3)	422 (96.1)	
Yes	82 (4.7)	29 (6.7)	19 (4.6)	17 (3.7)	17 (3.9)	
Previous stroke, *n* (%)						0.845
No	1,610 (92.5)	398 (91.5)	383 (92.7)	421 (92.7)	408 (92.9)	
Yes	131 (7.5)	37 (8.5)	30 (7.3)	33 (7.3)	31 (7.1)	
DM, *n* (%)						0.012
No	1,567 (90.0)	394 (90.6)	382 (92.5)	413 (91)	378 (86.1)	
Yes	174 (10.0)	41 (9.4)	31 (7.5)	41 (9)	61 (13.9)	
Day_of_illness, days	30.4 (15.0, 70.0)	36.0 (16.3, 75.5)	30.0 (15.0, 66.4)	31.7 (15.0, 72.0)	23.0 (11.0, 60.8)	< 0.001
BG, *n* (%)						0.728
No	769 (44.2)	200 (46)	186 (45)	194 (42.7)	189 (43.1)	
Yes	972 (55.8)	235 (54)	227 (55)	260 (57.3)	250 (56.9)	
BS, *n* (%)						0.442
No	1,509 (86.7)	384 (88.3)	353 (85.5)	387 (85.2)	385 (87.7)	
Yes	232 (13.3)	51 (11.7)	60 (14.5)	67 (14.8)	54 (12.3)	
CB, *n* (%)						0.373
No	1,662 (95.5)	414 (95.2)	391 (94.7)	440 (96.9)	417 (95)	
Yes	79 (4.5)	21 (4.8)	22 (5.3)	14 (3.1)	22 (5)	
Ischemic stroke, *n* (%)						0.734
No	501 (28.8)	134 (30.8)	115 (27.8)	126 (27.8)	126 (28.7)	
Yes	1,240 (71.2)	301 (69.2)	298 (72.2)	328 (72.2)	313 (71.3)	
ALB (g/L)	40.1 ± 3.9	35.2 ± 2.1	38.8 ± 0.6	41.1 ± 0.8	45.1 ± 2.1	< 0.001
FMA	54.5 ± 30.9	47.5 ± 30.2	54.0 ± 30.8	54.9 ± 30.4	61.3 ± 31.0	< 0.001
ADL	58.0 ± 27.4	47.3 ± 26.9	56.2 ± 27.0	60.8 ± 25.0	67.4 ± 26.7	< 0.001
BBS	23.0 (5.0, 43.0)	10.0 (2.5, 32.5)	21.0 (5.0, 42.0)	27.0 (6.0, 43.8)	35.0 (9.0, 48.5)	< 0.001
MoCA	19.4 ± 8.1	17.2 ± 8.4	18.8 ± 8.1	20.2 ± 7.1	21.2 ± 8.0	< 0.001

CAD, Coronary heart disease; DM, Diabetes mellitus; BG, Basal ganglia; BS, Brainstem; CB, Cerebellum; FMA, Fugl-Meyer Assessment of Motor Function; ADL, Activities of Daily Living; BBS, Berg Balance Scale; MoCA, Montreal Cognitive Assessment.

### Association between ALB levels and functional outcomes

Table 2 summarizes the results of the multiple linear regression analysis examining the association of ALB with FMA, ADL, BBS, and MoCA scores. The analysis included three models, with Model 3 being maximally adjusted for sex, age, education, smoking, drinking, BMI, hypertension, DM, CAD, previous stroke, Day_of_illness, and lesion locations (BG, BS, and CB). ALB levels exhibited significant positive associations with all functional outcomes across incremental adjustment models (Table 2). In the fully adjusted Model 3, ALB, analyzed as a continuous variable (per 1 g/L increase), was significantly associated with higher scores in FMA (*β* = 1.35, 95% CI: 0.99 ~ 1.72, *p* < 0.001), ADL (*β* = 1.77, 95% CI: 1.44 ~ 2.1, p < 0.001), BBS (*β* = 1.02, 95% CI: 0.78 ~ 1.26, *p* < 0.001), and MoCA (β = 0.30, 95% CI: 0.21 ~ 0.40, *p* < 0.001). When ALB was analyzed categorically (quartiles), a significant dose-dependent relationship was observed for all outcomes. Patients in the highest ALB quartile (≥42.8 g/L) exhibited markedly higher FMA (*β* = 15.11, 95% CI: 11.09–19.12), ADL (β = 19.35, 95% CI: 15.76–22.93), BBS (β = 11.61, 95% CI: 9.00–14.22), and MoCA scores (β = 3.12, 95% CI: 2.07–4.17) compared to the lowest quartile (all *p* < 0.001). Trend tests confirmed progressively stronger functional improvements across ascending ALB quartiles (all *p* < 0.001). Associations remained robust after adjusting for demographics and clinical covariates.

**Table 2:** Multiple linear regression analysis of factors associated with ALB and functional outcomes (FMA, ADL, BBS, and MoCA).

Variable	ALB (g/L)	*N* total	Model 1		Model 2		Model 3	
			Coefficient (95%CI)	*p* value	Coefficient (95%CI)	*p* value	Coefficient (95%CI)	*p* value
FMA	ALB (g/L)	1741	1.24 (0.88 ~ 1.61)	<0.001	1.37 (0.99 ~ 1.76)	<0.001	1.35 (0.99 ~ 1.72)	<0.001
	< 37.7 g/L	435	0(Ref)		0(Ref)		0(Ref)	
	37.7–40 g/L	413	6.59 (2.47 ~ 10.71)	0.002	7.05 (2.96 ~ 11.15)	0.001	6.89 (2.96 ~ 10.82)	0.001
	40–42.8 g/L	454	7.41 (3.39 ~ 11.43)	<0.001	8.50 (4.44 ~ 12.56)	<0.001	8.84 (4.94 ~ 12.74)	<0.001
	≥ 42.8 g/L	439	13.90 (9.84 ~ 17.95)	<0.001	15.02 (10.84 ~ 19.20)	<0.001	15.11 (11.09 ~ 19.12)	<0.001
	*P* for trend			<0.001		<0.001		<0.001
ADL	ALB (g/L)	1741	1.86 (1.54 ~ 2.17)	<0.001	1.75 (1.42 ~ 2.08)	<0.001	1.77 (1.44 ~ 2.10)	<0.001
	< 37.7 g/L	435	0(Ref)		0(Ref)		0(Ref)	
	37.7–40 g/L	413	8.87 (5.31 ~ 12.43)	<0.001	8.66 (5.10 ~ 12.22)	<0.001	8.90 (5.39 ~ 12.41)	<0.001
	40–42.8 g/L	454	13.52 (10.04 ~ 16.99)	<0.001	12.95 (9.42 ~ 16.47)	<0.001	13.36 (9.88 ~ 16.84)	<0.001
	≥ 42.8 g/L	439	20.07 (16.56 ~ 23.57)	<0.001	18.85 (15.22 ~ 22.48)	<0.001	19.35 (15.76 ~ 22.93)	<0.001
	*P* for trend			<0.001		<0.001	6.26 (5.12 ~ 7.40)	<0.001
BBS	ALB (g/L)	1741	1.13 (0.9 ~ 1.36)	<0.001	1 (0.76 ~ 1.24)	<0.001	1.02 (0.78 ~ 1.26)	<0.001
	< 37.7 g/L	435	0(Ref)		0(Ref)		0(Ref)	
	37.7–40 g/L	413	5.86 (3.28 ~ 8.45)	<0.001	5.69 (3.11 ~ 8.26)	<0.001	5.85 (3.29 ~ 8.40)	<0.001
	40–42.8 g/L	454	8.52 (5.99 ~ 11.04)	<0.001	7.95 (5.39 ~ 10.50)	<0.001	8.17 (5.63 ~ 10.70)	<0.001
	≥ 42.8 g/L	439	12.56 (10.02 ~ 15.11)	<0.001	11.28 (8.65 ~ 13.91)	<0.001	11.61 (9.00 ~ 14.22)	<0.001
	*P* for trend			<0.001		<0.001		<0.001
MoCA	ALB (g/L)	1741	0.39 (0.29 ~ 0.48)	<0.001	0.30 (0.21 ~ 0.40)	<0.001	0.30 (0.21 ~ 0.40)	<0.001
	< 37.7 g/L	435	0(Ref)		0(Ref)		0(Ref)	
	37.7–40 g/L	413	1.64 (0.57 ~ 2.7)	0.003	1.58 (0.55 ~ 2.61)	0.003	1.52 (0.49 ~ 2.54)	0.004
	40–42.8 g/L	454	3.00 (1.96 ~ 4.04)	<0.001	2.64 (1.63 ~ 3.66)	<0.001	2.61 (1.59 ~ 3.62)	<0.001
	≥ 42.8 g/L	439	4.00 (2.95 ~ 5.05)	<0.001	3.15 (2.11 ~ 4.20)	<0.001	3.12 (2.07 ~ 4.17)	<0.001
	*P* for trend			<0.001		<0.001		<0.001

Model 1: unadjusted. Model 2: adjusted for sex, age, education, smoke, drink, BMI, hypertension, DM, CAD, Previous stroke. Model 3: adjusted for all covariates in model 2 plus Day_of_illness, BG, basal ganglia; BS, brainstem; CB, cerebellum.

The figure displays the coefficients (β) and 95% confidence intervals (95% CI) representing the change in functional score per 1 g/L increase in serum ALB, stratified by predefined baseline characteristics. Each subgroup analysis was conducted using a multiple linear regression model (similar to Model 3) where the association between ALB and the outcome was assessed within each stratum. The models were comprehensively adjusted for all covariates, excluding the stratification factor itself.

### Subgroup analysis of the association between ALB levels and functional outcome

**Figure 2:**
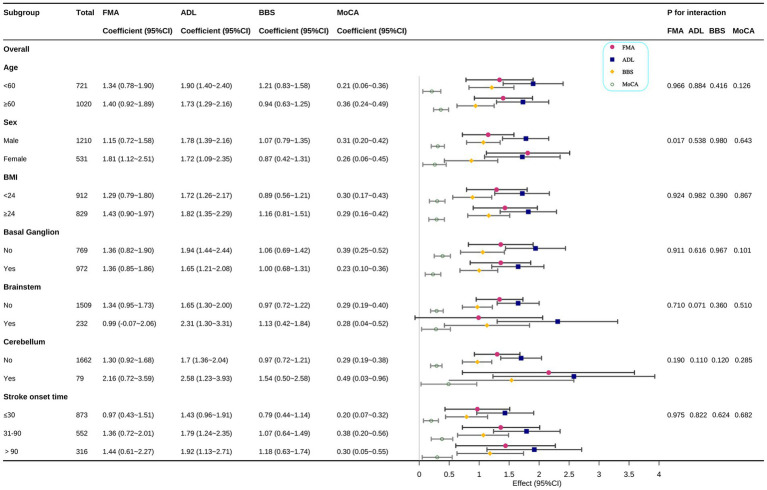
Subgroup analysis of the association between ALB and comprehensive functional outcomes (FMA, ADL, BBS, and MoCA).

The stability of the independent association between ALB (analyzed as a continuous variable per 1 g/L increase) and the four functional outcomes (FMA, ADL, BBS, and MoCA) was evaluated across predefined clinical subgroups, with the results presented in Figure 2.

Subgroup analyses revealed significant effect modification by sex for FMA (*p* = 0.017), with a stronger albumin-FMA association in females (*β* = 1.81, 95% CI: 1.12–2.51) than males (β = 1.15, 0.72–1.58). Overall, the positive association between higher ALB levels and better functional outcomes remained consistent (β > 0) across most subgroups defined by Age (< 60 vs. age ≥60 years), BMI (< 24 vs. ≥24 kg/m^2^), stroke onset time and lesion location (Basal Ganglion, Brainstem, and Cerebellum presence). No Significant Interaction for Other Subgroups. The association of ALB with FMA, ADL, BBS, and MoCA scores did not differ significantly by age, BMI, stroke onset time or the presence of lesions in the basal ganglion, brainstem, or cerebellum.

## Discussion

The current multicenter cross-sectional study systematically investigated the independent association between ALB levels and comprehensive functional deficits in a large cohort of 1,741 Chinese post-stroke patients. Our primary findings demonstrate a robust, independent, and dose-dependent positive association between higher serum ALB levels and better functional outcomes across all four critical domains: motor recovery (FMA), independence in daily activities (ADL), balance function (BBS), and cognitive status (MoCA). In the maximally adjusted Model 3, Each 1-g/L ALB increment in ALB was independently associated with significant improvements in functional gains (FMA: *β* = 1.35; ADL: β = 1.77; BBS: β = 1.02; MoCA: β = 0.3; all *p* < 0.001). Furthermore, patients in the highest ALB quartile (Q4, ≥42.8 g/L) achieved significantly higher scores across all outcomes compared to those in the lowest quartile (Q1, <37.7 g/L). Sex-specific effect modification was observed, with females showing a 57% stronger ALB-FMA association than males (β = 1.81 vs. 1.15; P interaction = 0.017), though no sex interaction was observed for other domains. This suggests potential biological or physiological differences in how nutritional status, inflammation, and stroke recovery interact between genders (20). Possible explanations include differences in body composition (e.g., lower lean muscle mass in females making them more susceptible to nutritional deficits), differential inflammatory responses, or variations in estrogen-mediated neuroprotection. This finding warrants further investigation through longitudinal studies.

The positive association between higher serum ALB levels and better functional outcomes was found to be robust and consistent across all tested subgroups defined by age (< 60 vs. ≥ 60 years), BMI (< 24 vs. ≥ 24 kg/m^2^), stroke onset time and primary lesion locations (BG, BS, and CB). Although not statistically significant, there was a trend toward an amplified effect of ALB on functional outcomes in patients with cerebellar lesions. While further longitudinal data are required to confirm this finding, it suggests that greater attention should be paid to rehabilitation and nutritional strategies for patients with cerebellar lesions. These results underscore the potential of ALB as a crucial and readily available biomarker for predicting the full spectrum of post-stroke functional impairment.

The observed association aligns with and significantly extends existing literature, which predominantly focused on single, often less objective, outcomes. The finding that hypoalbuminemia is detrimental to overall recovery is consistent with studies linking low ALB to poor functional prognosis, typically assessed by the modified Rankin Scale (mRS) or mortality (10–12, 21). While previous reports have suggested a link between low ALB and poor motor function or cognitive decline, our study provides the first large-scale evidence confirming this link simultaneously across four distinct, validated functional domains in an Asian cohort, after rigorous adjustment for key confounders. Notably, the association with ADL (*β* = 1.77) and FMA (β = 1.35) showed the largest coefficients, indicating ALB status exerts a substantial influence on physical independence.

The observed robust association between ALB levels and multidomain functional recovery is supported by several plausible and multifactorial biological mechanisms. Firstly, low ALB serves as a primary marker of nutritional deficit and protein-energy wasting, which accelerates muscle catabolism and precipitates sarcopenia. This systemic wasting directly impedes physical recovery, negatively affecting motor function (FMA) and overall physical performance (ADL/BBS) (12). Secondly, albumin functions as a potent negative acute-phase reactant and antioxidant (22). Consequently, hypoalbuminemia reflects heightened systemic inflammation following a stroke. This chronic inflammatory state is detrimental, known to impair essential neuroplasticity and recovery processes, thereby contributing to poorer outcomes across all functional domains, including cognition. Finally, ALB plays a critical role in maintaining plasma oncotic pressure. Severe hypoalbuminemia may thus compromise blood–brain barrier integrity and potentially exacerbate cerebral edema, which collectively contribute to negative impacts on brain tissue recovery and long-term function (11, 21).

Our findings indicate that ALB may serve as a valuable hematological biomarker that can assist rehabilitation physicians and therapists in comprehensively assessing patients and implementing targeted interventions following a stroke. This assertion is further corroborated by evidence suggesting that low serum ALB levels are significantly associated with long-term depressive symptoms in older stroke survivors (23). Considering the substantial burden of post-stroke functional impairment in China, which severely impacts patients’ quality of life (24), our results point toward a critical need for early identification and intervention. By prioritizing the monitoring of ALB levels, clinicians can proactively identify nutritional risk and implement rehabilitation interventions sooner. This strategy represents a paradigm shift, achieving a “front-loading” of risk identification via laboratory indicators. We emphasize that future research should focus on integrating standardized nutritional assessments with functional outcomes to maximize benefits for the stroke population.

### Strengths and limitations

The major strengths of this study include the large sample size (N = 1741) drawn from multiple centers, enhancing the external validity of our findings. Crucially, we employed highly specific, validated measures for each functional domain (FMA, BBS, MoCA, ADL), moving beyond single gross disability scores. Furthermore, the inclusion of a comprehensive set of covariates, including lesion location (BG, BS, CB) and Day_of_illness, allowed us to establish the independent association of ALB, minimizing residual confounding. The demonstration of a robust dose-dependent relationship across ALB quartiles strengthens the causal inference. Despite its strengths, this study has several limitations inherent to its cross-sectional design. First, the cross-sectional nature precludes the establishment of temporal or causal relationships; while we adjusted for time since stroke, reverse causality (i.e., severe disability leading to poor nutritional intake and low ALB) cannot be entirely excluded. Second, ALB levels reflect both nutritional status and inflammation; we did not adjust for specific inflammatory markers (e.g., CRP), which could introduce residual confounding. Third, the cohort was derived from rehabilitation centers, which may introduce selection bias towards patients eligible and stable enough for intensive rehabilitation.

## Conclusion

In summary, this study provides evidence that serum ALB levels are independently and dose-dependently associated with multidomain functional status—including motor, ADL, balance, and cognitive function—within a Chinese post-stroke rehabilitation cohort. Our analysis indicates that higher ALB levels, particularly within the highest quartile (≥42.8 g/L), correlate with superior functional outcomes, whereas lower levels (specifically < 37.7 g/L) are linked to poorer performance. Furthermore, the correlation between ALB and motor recovery exhibits sex-specific patterns, appearing more pronounced in female patients. These findings highlight the potential of ALB as an objective biological indicator for risk stratification and the assessment of rehabilitation potential. Future longitudinal studies and randomized controlled trials are warranted to further investigate these associations and determine whether maintaining optimal ALB levels can improve functional recovery after stroke.

## Data Availability

The raw data supporting the conclusions of this article will be made available by the authors, without undue reservation.

## References

[1] GBD 2023 Causes of Death Collaborators. Global burden of 292 causes of death in 204 countries and territories and 660 subnational locations, 1990-2023: a systematic analysis for the global burden of disease study 2023. Lancet. (2025) 406:1811–72. doi: 10.1016/S0140-6736(25)01917-8 41092928 PMC12535838

[2] Tu W-J Wang L-D. China stroke surveillance report 2021. Mil Med Res. (2023) 10:33. doi: 10.1186/s40779-023-00463-x, 37468952 PMC10355019

[3] Ma Q Li R Wang L Yin P Wang Y Yan C . Temporal trend and attributable risk factors of stroke burden in China, 1990-2019: an analysis for the global burden of disease study 2019. Lancet Public Health. (2021) 6:e897–906. doi: 10.1016/S2468-2667(21)00228-0, 34838196 PMC9047702

[4] Zhao Y Hua X Ren X Ouyang M Chen C Li Y . Increasing burden of stroke in China: a systematic review and meta-analysis of prevalence, incidence, mortality, and case fatality. Int J Stroke. (2023) 18:259–67. doi: 10.1177/17474930221135983, 36274585

[5] Skidmore ER Eskes G Brodtmann A. Executive function Poststroke: concepts, recovery, and interventions. Stroke. (2023) 54:20–9. doi: 10.1161/STROKEAHA.122.037946, 36542071

[6] Ullberg T Zia E Petersson J Norrving B. Changes in functional outcome over the first year after stroke: an observational study from the Swedish stroke register. Stroke. (2015) 46:389–94. doi: 10.1161/STROKEAHA.114.006538, 25538204

[7] Arques S. Human serum albumin in cardiovascular diseases. Eur J Intern Med. (2018) 52:8–12. doi: 10.1016/j.ejim.2018.04.014, 29680174

[8] Allison SP Lobo DN. The clinical significance of hypoalbuminaemia. Clin Nutr. (2024) 43:909–14. doi: 10.1016/j.clnu.2024.02.018, 38394971

[9] Gremese E Bruno D Varriano V Perniola S Petricca L Ferraccioli G. Serum albumin levels: a biomarker to be repurposed in different disease settings in clinical practice. J Clin Med. (2023) 12:6017. doi: 10.3390/jcm12186017, 37762957 PMC10532125

[10] Mehta A De Paola L Pana TA Carter B Soiza RL Kafri MW . The relationship between nutritional status at the time of stroke on adverse outcomes: a systematic review and meta-analysis of prospective cohort studies. Nutr Rev. (2022) 80:2275–87. doi: 10.1093/nutrit/nuac034, 35640017 PMC9647329

[11] Thuemmler RJ Pana TA Carter B Mahmood R Bettencourt-Silva JH Metcalf AK . Serum albumin and post-stroke outcomes: analysis of UK regional registry data, systematic review, and Meta-analysis. Nutrients. (2024) 16:1486. doi: 10.3390/nu16101486, 38794724 PMC11124370

[12] Bi R Shi Y Li M Liu X Ma Z Huang Y . Association between serum albumin and severe impairment of activities of daily living in patients with stroke: a cross-sectional study. Front Neurol. (2025) 15:1501294. doi: 10.3389/fneur.2024.1501294, 39835151 PMC11743378

[13] von Elm E Altman DG Egger M Pocock SJ Gøtzsche PC Vandenbroucke JP. The strengthening the reporting of observational studies in epidemiology (STROBE) statement: guidelines for reporting observational studies. Lancet. (2007) 370:1453–7. doi: 10.1016/S0140-6736(07)61602-X, 18064739

[14] Li X Cao X Ying Z Zhang J Sun X Hoogendijk EO . Associations of serum albumin with disability in activities of daily living, mobility and objective physical functioning regardless of vitamin D: cross-sectional findings from the Chinese longitudinal healthy longevity survey. Front Nutr. (2022) 9:809499. doi: 10.3389/fnut.2022.809499, 35284431 PMC8908380

[15] Gladstone DJ Danells CJ Black SE. The fugl-meyer assessment of motor recovery after stroke: a critical review of its measurement properties. Neurorehabil Neural Repair. (2002) 16:232–40. doi: 10.1177/154596802401105171, 12234086

[16] Berg K Wood-Dauphinee S Williams JI. The balance scale: reliability assessment with elderly residents and patients with an acute stroke. Scand J Rehabil Med. (1995) 27:27–36. doi: 10.2340/1650197719952736, 7792547

[17] Wei X Ma Y Wu T Yang Y Yuan Y Qin J . Which cutoff value of the Montreal cognitive assessment should be used for post-stroke cognitive impairment? A systematic review and meta-analysis on diagnostic test accuracy. Int J Stroke. (2023) 18:908–16. doi: 10.1177/17474930231178660, 37190789

[18] Nasreddine ZS Phillips NA Bédirian V Charbonneau S Whitehead V Collin I . The Montreal cognitive assessment, MoCA: a brief screening tool for mild cognitive impairment. J Am Geriatr Soc. (2005) 53:695–9. doi: 10.1111/j.1532-5415.2005.53221.x, 15817019

[19] Swartz RH Longman RS Lindsay MP Lund R Ganesh A Eskes GA . Canadian stroke best practice recommendations: vascular cognitive impairment, 7th edition practice guidelines update, 2024. Alzheimers Dement. (2025) 21:e14324. doi: 10.1002/alz.14324, 39822128 PMC11772713

[20] Rexrode KM Madsen TE Yu AYX Carcel C Lichtman JH Miller EC. The impact of sex and gender on stroke. Circ Res. (2022) 130:512–28. doi: 10.1161/CIRCRESAHA.121.319915, 35175851 PMC8890686

[21] Zhu Y Xue G Xu S Qin Q Liu P Ji L . U-shaped relationship of serum albumin and neurological functional outcomes after acute ischemic stroke: a prospective cohort study. Neurol Ther. (2025) 14:949–64. doi: 10.1007/s40120-025-00729-7, 40237930 PMC12089567

[22] Wang Y-Q He X Huang X-L Qin F-L Mao F Cheng Y-M . The role of serum albumin and albumin-related nutritional indices in predicting post-stroke cognitive impairment: a systematic review and meta-analysis. Front Neurol. (2025) 16:1641711. doi: 10.3389/fneur.2025.1641711, 40881792 PMC12382350

[23] Pascoe MC Skoog I Blomstrand C Linden T. Albumin and depression in elderly stroke survivors: an observational cohort study. Psychiatry Res. (2015) 230:658–63. doi: 10.1016/j.psychres.2015.10.023, 26520562

[24] Zhao P Sun H. Evaluating the rehabilitation needs of stroke patients in China: a trend analysis from 1990 to 2019. Brain Behav. (2025) 15:e70389. doi: 10.1002/brb3.70389, 40079486 PMC11904947

